# The Medical Profession in California

**Published:** 1875-06

**Authors:** 


### Editorial.

### The Medical Profession in California.

The San Francisco *News Letter*, a weekly paper, began some months since to publish from week to week, a list of quacks, including with the most notorious charlatans a number of respectable practitioners.

The *Pacific Medical and Surgical Journal* has until the present time seen fit to say nothing about the subject, preferring to let the matter alone and watch developments. The recent action of the *London Lancet* and the *Boston Medical and Surgical Journal*, in giving credence to these stories, has, however, called forth a reply from the Editors. They boldly assert, that the *News Letter* published the list and statements for purely black-mailing purposes, and state that several physicians, whose names were included in the list, had them removed by paying sums of money.

We are sorry that a professional journal would extend this slander without inquiring into the facts more particularly, but we have no doubt that the Editors of the *Lancet and Boston Medical Journal* will both hasten to make ample apology, on being informed of the untruthfulness of the charges to which they have given publicity.

The difficulty seems to have been increased by certain members of the medical profession, who, we are sorry to say, from selfish and malign motives, sought to aid the *News Letter* in slandering, and took the occasion to avenge themselves upon members of the profession who had incurred their enmity. It is a sad truth, that such men can be found in the ranks of what should be in every sense a learned and liberal profession, but if the San Francisco physicians can, by this trial, rid themselves of a few of them, they will be amply repaid for the slander which has been circulated against them. They have our sympathy and best wishes for a triumphant vindication of the right.

